# Evaluation of triply periodic minimal surface geometries in 3D‐printed PLA scaffolds for chondrogenic differentiation

**DOI:** 10.1002/btpr.70062

**Published:** 2025-08-11

**Authors:** Mahmut Alp Kılıç, Mustafa Akyürek, Roozbeh Abidnejad, Alp Karakoç

**Affiliations:** ^1^ Faculty of Medicine, Department of Biophysics Aydin Adnan Menderes University Aydin Turkey; ^2^ Faculty of Medicine Department of Plastic Reconstructive and Aesthetic Surgery Canakkale Onsekiz Mart University Canakkale Turkey; ^3^ Department of Bioproducts and Biosystems School of Chemical Engineering, Aalto University Espoo Finland; ^4^ Department of Information and Communications Engineering Aalto University Espoo Finland

**Keywords:** cartilage, chondrogenic differentiation, porous scaffolds, tissue engineering, triply periodic minimal surface (TPMS)

## Abstract

Triply periodic minimal surface (TPMS) scaffolds are gaining attention in tissue engineering due to their continuous and interconnected porous architecture. In this study, three TPMS geometries—Gyroid, Diamond, and I‐WP—were fabricated from polylactic acid (PLA) using fused deposition modeling (FDM), with all scaffolds designed to maintain the same overall porosity. Scaffold characterization included scanning electron microscopy (SEM), microcomputed tomography (micro‐CT), compressive mechanical testing, and surface wettability analysis. Although porosity was constant, differences in Equivalent Circular Diameter (ECD) values were observed among the geometries, reflecting variations in pore morphology. Adipose‐derived stem cells (ADSCs) were seeded onto the scaffolds and cultured under chondrogenic differentiation conditions for 21 days. Cell viability, gene expression (*Col2, Col10, Sox9*), and protein levels were assessed using RT‐PCR and Western blot. All scaffold geometries supported cell attachment and chondrogenic differentiation to varying degrees. The Diamond geometry showed the highest chondrogenic marker expression at the mRNA level, while the Gyroid geometry promoted more stable protein expression with reduced hypertrophic signaling. These findings demonstrate that scaffold geometry, even under identical material and porosity conditions, can influence stem cell behavior. The results offer valuable insights for optimizing TPMS‐based scaffold designs in cartilage tissue engineering applications.

## 1 INTRODUCTION

Tissue engineering, a subfield of regenerative medicine, not only offers the potential to develop transplantable tissues and organs but also includes new experimental pathways for treating various diseases.^1^, ^2^, ^3^ Among modern regenerative medicine applications, the use of cell scaffolds appears to be an important tool in developing various tissue types. When these scaffolds are seeded with suitable cells and successful colonization occurs, they can be used as suitable bioimplants that can be transplanted into the living. The type of material used in purpose‐built cell scaffolds, the geometry and pore structure of the scaffold are found to be important factors in achieving the desired goal.^4^, ^5^, ^6^, ^7^ For a scaffold to be effective, it must have appropriate mechanical stability and properties that will ensure biocompatibility and withstand environmental stressors during seeding and after implantation into the body.^8^, ^9^, ^10^

Due to its natural characteristics, such as low vascularization and innervation, the limited regeneration capacity of cartilage tissue presents a challenge in tissue engineering applications as well as in tissue healing.^11^ For example, when chondrocyte cells are cultured on flat surfaces in vitro, they lose their ability to synthesize essential proteins for hyaline cartilage formation.^12^, ^13^ In contrast, complex porous cell scaffolds with interconnected macropores are potential candidates for a solution. Such scaffold structures not only increase the cell seeding surface but also support cell migration, development, and tissue maturation.^9^, ^14^ Although several studies have shown that such scaffolds can enhance the regenerative potential of cartilage tissue, the magnitude of this potential may depend on the design and structure of the scaffolds (Kwon et al., 2019^11^, ^15^, ^16^;). This includes not only having the right architectural design but also features such as controlled porosity and biocompatibility. Additionally, it is very important that these scaffolds exhibit sufficient mechanical strength and actively support cellular functions such as adhesion, migration, and development. Looking deeper into the design details, elements such as pore size, interconnectivity, permeability, and the type of material used stand out as critical determinants of how successfully these scaffolds can facilitate cartilage regeneration.^4^, ^6^

Detailed examination of microstructures such as bone and cartilage, where matrix geometry and physical properties play a major role, shows that these structures have properties like specific organizations called three‐dimensional periodic minimal surfaces (TPMS). TPMSs are a type of periodic covered surfaces with zero mean curvature; the porous structures they form are smooth, and their lattice structures do not have sharp edges and connection points. Another advantage of the porous structures formed by these geometries is that they have highly interconnected, low‐curvature inner surfaces. These properties have greatly benefited a wide range of scientific and engineering applications including heat exchangers, flow and mass transfer solutions, structural damping, and acoustic barriers, to name a few.^17^, ^18^, ^19^ Interestingly, current literature studies also demonstrate that cell scaffolds using TPMS surfaces can support differentiation toward native tissues with similar geometries such as bone or cartilage.^20^, ^21^ Moreover, it has been shown before that small differences in surface topography and matrix geometry can influence stem cell behavior, directing cell aggregation and differentiation.^22^, ^23^

In contribution to these studies on the tissue and scaffold engineering, we aimed to evaluate the effectiveness of three TPMS geometries, i.e. Diamond, Gyroid, and I‐WP, on the chondrogenic differentiation of adipose stem cells in vitro. For this purpose, three different polylactic acid (PLA) groups with a well‐established record of success in biomedicine were used to additively manufacture these geometries. Prior to performing biological evaluations, we characterized the printed scaffold geometries with micro‐computed tomography (micro‐CT) to analyze their internal architecture and porosity. We also carried out compressive strength testing to assess mechanical performance and used scanning electron microscopy (SEM) to investigate surface morphology. Furthermore, we conducted surface hydrophilicity tests to evaluate wettability, as this factor is recognized to influence cell attachment and behavior. Then, adipose stem cells (ADSCs) were seeded on each geometry, and chondrogenic differentiation was initiated. After 21 days of in vitro differentiation, chondrogenic differentiation marker levels on scaffold geometry were evaluated between groups by RT‐PCR and western blot methods.

## 2 MATERIALS AND METHODS

### 2.1 Triply periodic minimal surface scaffold design and manufacture

The generation of triply periodic minimal surface (TPMS) scaffold print functions followed a systematic approach to ensure precise design and porosity control. Three different TPMS structures, including Diamond, Gyroid, and I‐WP surfaces, were mathematically represented as nodal equations, as detailed in a previous study and listed in Table 1, to define their geometric and topological properties.^24^ Finalized designs were exported as STL files, ensuring compatibility with 3D printing software and machines. All Mathematica notebooks and sample STL files are publicly accessible at https://github.com/metudust/RegionTPMS, along with detailed documentation to assist users in customizing TPMS scaffolds.

**TABLE 1:** The table below presents the mathematical formulations for three iconic minimal surfaces: The Diamond, Gyroid, and I‐WP (Infinite Periodic Minimal Surface). These surfaces are described using trigonometric equations that characterize their periodic structures and intrinsic symmetries.

Diamond	sin(*x*) sin(*y*) sin(*z*) + sin(*x*) cos(*y*) cos(*z*) + cos(*x*) sin(*y*) cos(*z*) + cos(*x*) cos(*y*) sin(*z*)
Gyroid	cos(*x*) sin(*y*) + sin(*x*) cos(*z*) + cos(*y*) sin(*z*)
I‐WP	2 (cos(*x*) cos(*y*) + cos(*x*) cos(*z*) + cos(*y*) cos(*z*)) − (cos(2*x*) + cos(2*y*) + cos(2*z*))

Thereafter, the scaffold designs were fabricated with fused deposition modeling (FDM) technique, which is an affordable yet precise manufacturing solution (Figure S1). All scaffolds were designed with a target porosity of 0.2 (20%) prior to fabrication. For this matter, three different polymer filaments were employed. We have used PLA (polylactic acid) and PVA (Polyvinyl alcohol) filaments to fabricate the main structure and the support structure, respectively. Regarding the PLA, we first used XPLA provided by add: north company (Stockholm, Sweden). The XPLA is manufactured utilizing entirely biodegradable material and is reported to have a ca. 1.24 g/cm^3^ density and a glass transition temperature (Tg) of 55°C. The XPLA filament diameter is reported to be 2.85 ± 0.05 mm. The second PLA used in this study was Tough‐PLA provided by Ultimaker company (through an official retailer in Finland). Tough PLA was reported to have a density of 1.22 g/cm^3^, a Tg of 59°C, and a filament diameter of 2.85 ± 0.05 mm. Regarding the support structure, we were provided with water‐soluble PVA filament by the Ultimaker company (through an official retailer in Finland). The 3D printing process was conducted via the Ultimaker 3 extended dual 3D printer (Ultimaker, Netherlands). For both PLAs, the nozzle temperature was set to be 210°C and the bed temperature was 60°C. For PVA, the nozzle temperature was taken to be 225°C. The fabrication process comprised synchronized dual printing so that print cores AA 0.4 and BB 0.4 mm were used for PLA and PVA, respectively. As shown in Figure S1, the PVA support was printed inside and outside the main structure to support the printing process while the height was increasing. After finalizing the printing process, samples were detached from the printing bed, placed inside a deionized water, and subjected to stirring process for 12 hours. During every 12 hours, the water was changed until there was no PVA residue. Finally, the specimens were subjected to ambient evaporation for 24 hours.

### 2.2 Morphological and structural characterization

The surface morphologies of Diamond, Gyroid, and I‐WP scaffolds were analyzed using scanning electron microscopy (SEM) with a Zeiss Sigma VP electron microscope equipped with a Schottky field emission source. The imaging was performed at an electron high tension (EHT) of 1.5 kV to minimize charging effects and preserve surface details. Prior to the imaging, the samples were coated with a 4 nm layer of platinum/palladium alloy using a Leica EM ACE600 sputter coater under high vacuum conditions. This thin conductive coating ensured optimal imaging quality by reducing the electron charging. Representative images were captured to analyze the surface features and ensure detailed characterization of the scaffold surface structure and porosity.

Thereafter, cell scaffolds were analyzed using a high‐resolution micro‐computed tomography (micro‐CT) scanner (SkyScan 1172, Bruker, Belgium) at 85 kV and 118 μA with an Al + Cu filter. Scanning used an 11 MP Hamamatsu camera in 2 × 2 binning mode and 2300 ms exposure per projection. Reconstruction via NRecon (v1.6.10.6, GPUReconServer engine) yielded a voxel size of 13.69 μm. Gaussian smoothing (kernel: 2), beam hardening correction (30%), and ring artifact correction (level 7) were applied. Final 16‐bit axial images (2000 × 2000 pixels) were saved in DICOM format. Porosity was assessed using cross‐sectional micro‐CT slices binarized to separate solid and void areas. A consistent region of interest (ROI) was applied across slices. ImageJ measured pore and scaffold areas in each ROI, and porosity was calculated as the ratio of total pore area to total scaffold area. Pore diameter was estimated using the Equivalent Circular Diameter (ECD), derived from binarized images via the Analyze Particles function in ImageJ. Noise was excluded using a minimum size threshold. The mean pore diameter and standard deviation were then calculated.

Compression tests were performed to evaluate the mechanical properties of the scaffolds. Cylindrical samples with a diameter of 8 mm and a height of 3 mm were tested axially using a universal testing machine (Testform / AS1, Turkey) at a constant displacement rate of 1 mm/min. A 5 kN load cell was used for all measurements. Each geometry and porosity group was tested by using six specimens. The stress–strain curve and compressive strength were determined as the nominal values recorded during the test.

Furthermore, scaffold wettability was also analyzed by the static contact angle of specimens. ThetaFlex Optical Tensiometer from Biolin Scientific, Sweden was used for the evaluation purpose. The contact angles of samples were measured using a 5 μL droplet of Milli‐Q® water. The experiment lasted 120 seconds, and the statistics shown in the results represent the average of the final points gathered.

### 2.3 Cell culture

We utilized previously isolated and archived rat adipose stem cells from a prior study. The use of previously obtained primary cells was authorized by the'Operation, Procedures, and Principles of the Ethics Committees of Animal Experiment' as stipulated by the Republic of Turkey Ministry of Environment and Urbanization and was approved by the Aydın Adnan Menderes University Local Ethical Committee (ADU‐HADYEK 64583101/2024/60). The cells were stored in liquid nitrogen, thawed, and used according to the following protocol: Frozen cells were removed from liquid nitrogen and placed at −80°C for one day. The next day, the cells were thawed at 37°C, and the freezing medium, which contained 10% DMSO, was removed by centrifugation at 1000 rpm for 10 minutes at +4°C. After centrifugation, the cells were resuspended in a complete cell medium (α‐MEM (Sigma‐Aldrich) + 5% L‐glutamine (Sigma‐Aldrich) + 5% Penicillin–Streptomycin (Sigma‐Aldrich) + 10% FBS (Gibco Life Technologies)) and seeded in T75 flasks. The cell media were changed three times a week, and the cells were passaged when the cell density reached 80%.

### 2.4 Scaffold seeding and chondrogenic differentiation

The scaffolds underwent sterilization before cell transplantation, followed by a hydration and fibronectin coating process to enhance cell attachment. Initially, the scaffolds were placed in 50 mL luer lock syringes and immersed in 70% alcohol. To improve alcohol penetration into the scaffolds, the luer lock syringe plug was manipulated to create negative pressure, eliminating any air pockets within the scaffold. Subsequently, after a 2‐hour alcohol sterilization period, the alcohol was drained, and Hank's Balanced Salt Solution (HBSS) was introduced into the syringe, repeating the process five times. After HBSS washing, the same protocol was followed using HBSS solution containing 2 μg/mL fibronectin (PromoCell) for 24 hours at +4°C to allow for fibronectin permeation and coating of the scaffold. The following day, after a brief rinse with plain HBSS, a complete cell medium was introduced into the scaffold and allowed to permeate for 24 hours at +4°C.^25^ Following the coating and hydration preparations, the scaffolds, now containing the cell medium, were placed in sterile glass vials, and cells were seeded using the dynamic cell seeding method. Subsequently, 20 × 10^5^ cells were planted in 2 mL of cell medium. The vials containing the seeded scaffolds were then placed on an orbital shaker at 20 rpm and incubated at 37°C with 5% CO_2_ for 24 hours^26^ (Figure S2).

The efficacy of cell seeding was assessed using the CCK‐8 Cell Proliferation assay (Abcam) 24 hours after seeding. Cell‐laden scaffolds were incubated in 1 mL of complete cell medium containing 100 μL of CCK8 stock solution for 4 hours at 37°C and 5% CO_2_. Subsequently, absorbance was measured at 450 nm using a microplate reader. Cell seeding efficacy was calculated as the percentage of absorbance relative to 20 × 10^5^ cells seeded in a 24‐well plate the previous day. Values from the wells were analyzed as mean ± standard deviation. The efficiency of cell seeding was assessed by comparing the absorbance of cells present on the scaffold to the absorbance of those initially seeded in a 24‐well plate.

Following the seeding, the cell‐coated scaffolds were transferred to 24‐well cell plates with %2 agarose gel‐coated bottoms, 24 h later. To induce chondrocyte differentiation, each well was supplied with α‐MEM cell medium containing: insulin at a concentration of 6.25 μg/mL (Solarbio), transferrin at a concentration of 6.25 μg/mL (Sigma‐Aldrich), bovine serum albumin (BSA) at a concentration of 1.25 μg/mL (Sigma‐Aldrich), ascorbic acid at a concentration of 50 μg/mL (Solarbio), dexamethasone at a concentration of 10^−7^ M (Sigma‐Aldrich), and TGF‐β1 at a concentration of 10 ng/mL (Biolegend). Throughout the 21‐day duration, the differentiation medium was refreshed biweekly, according to the experimental protocol.^27^

### 2.5 Assessment of chondrogenic markers by real‐time PCR

Trizol RNA extraction method was used to examine the quantitative gene expression analysis of chondrocyte cell markers between experimental groups. After 21 days of differentiation, the cells in each scaffold were trypsinized. Following the manufacturer's instructions, the obtained cells were homogenized in trizol reagent, phase separated, sedimented, washed, and dissolved in RNAse‐free water. The total RNA concentration of the obtained samples was determined spectrophotometrically at 260/280 nm. For quantitative real‐time polymerase chain reaction (qRT‐PCR), we utilized SYBR Green qPCR Master mix and followed a reaction protocol consisting of an initial heating step at 95°C for 10 minutes, followed by 40 cycles of denaturation at 95°C for 15 seconds, annealing at 55°C for 10 minutes, and extension at 72°C for 30 seconds. The mRNA targets in our study included *Col1, Col2, Col10*, and *Sox9*, while *18 s* served as the housekeeping gene. The primer sequences for the genes analyzed in this study are as follows: For *Collagen I* (*Col1*), the forward primer is TGATCGATGCGATGGATGAG, and the reverse primer is TCGAACGATGCGAATGATGA. For *Collagen II* (*Col2*), the forward primer is AGAGACCTGAACTGGGCAGA, and the reverse primer is TGACACGGAGTAGCACCATC. For *Collagen X* (*Col10*), the forward primer is CCGTATGATACAGTAATGACT, and the reverse primer is TTGATGACAGTAGTACGTAT. The primers for *Sox 9* are TTCATGAAGATGACCGACGA (forward) and GTCCAGTCGTAGCCCTTGAG (reverse). Lastly, the primers for *18 s* are GGATAACGTACCTGATAG (forward) and ATTAGCCGTATGACTAGGAT (reverse). These primer sequences were used for amplification and subsequent analysis of the respective target genes. Fold differences were calculated using the ΔΔCT method. For each experimental group, RNA was extracted from a pooled sample of three scaffolds, and qRT‐PCR was performed in three independent replicates, each run in duplicate.

### 2.6 Assessment of Chondrogenic Markers by Western Blotting

Protein levels of Sox9 and Col2 are evaluated between the groups, which are genes prominently expressed by differentiated chondrogenic cells. In summary, cells were harvested and lysed in Western blot with %1 proteinase/phosphatase containing RIPA buffer (Thermo Scientific). Total protein concentrations were determined via the BCA kit (Takara). Equal amounts of proteins were denatured by heat and then subjected to SDS‐PAGE (stacking gel %4‐resolving gel %12) for 90 minutes at 110 V. Following the SDS‐PAGE, protein samples are transferred to a PVDF membrane. The transfer was verified by staining with Ponceau S dye. Then the membranes are incubated in T‐PBS containing 5% skim milk for 2 hours at room temperature to block non‐specific binding sites. Primary antibodies were applied to the membrane and incubated overnight at 4°C. This was followed by incubation with an HRP‐conjugated secondary antibody for 1 hour at room temperature. The membranes were then treated with the Pierce ECL Western blotting substrate kit (Thermo Scientific, MA, USA) for chemiluminescent detection of immunoreactive proteins. The primary antibodies used were a mouse anti‐rabbit collagen type II antibody (Chemicon, Temecula, CA) and an antibody against Sox9; all protein levels are normalized to the housekeeping protein GAPDH. For each sample, proteins were extracted from pooled lysates of 8–10 scaffolds, and two independent pooled samples per group were used for analysis.

## 3 STATISTICAL ANALYSIS

All data obtained were presented as mean ± standard error, except for scaffold characterization results, which were presented as mean ± standard deviation (SD). Statistical analyses were performed using GraphPad Prism version 10.4. After verifying the normal distribution of variables in the RT‐PCR and Western blot experiments, one‐way ANOVA was conducted for statistical evaluation, followed by Tukey's post hoc test for multiple comparisons. If normality was not met, the Kruskal‐Wallis test followed by Dunn's post hoc test was used. Statistical significance was set at *p* ≤ 0.05. The degree of significance, relative to the damage group, is denoted by an * = *p* < 0.05, ** = *p* < 0.01, *** = *p* < 0.001.

## 4 RESULTS

### 4.1 Characterization of TPMS scaffolds

**FIGURE 1:**
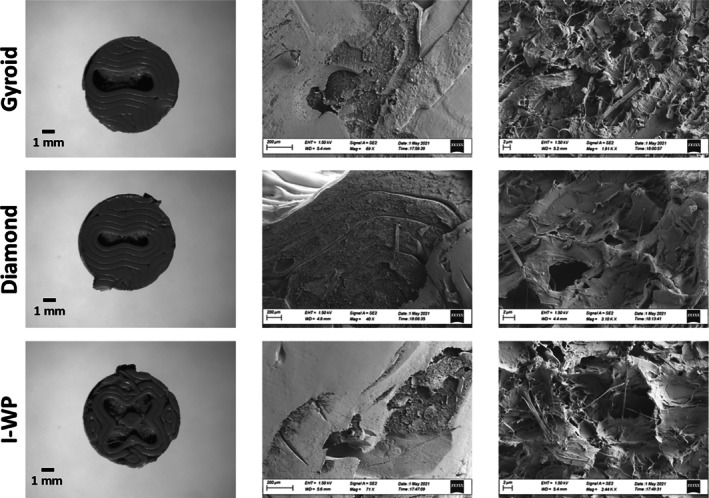
Macroscopic views of the scaffolds (left column) and corresponding Scanning Electron Microscopy (SEM) images (right column, scale bar = 2 μm), illustrating the surface morphology of three distinct scaffold designs. SEM analysis indicates that all scaffold types exhibit similar surface roughness and porosity characteristics at the microscale.

In Figure 1 the leftmost column clearly demonstrates the different printed geometries at a macroscopic level. In the middle column, the 200 μm scale SEM images of the same geometries provide an overview of the scaffold surfaces, showing the layered morphology formed during the printing process and macrostructural features such as cracks and smooth surfaces. On the right side of Figure 1, the 2 μm scale SEM images present a more detailed examination of the microstructural details, revealing a complex porous structure and a fibrous network. However, both the 200 μm and (especially) 2 μm SEM images reveal no significant differences between the scaffolds at the micron and submicron levels. These images demonstrate that all scaffold geometries retain similar micro‐ and submicron‐sized features after the 3D printing process, particularly exhibiting similar characteristics in terms of microstructural properties. Surface wettability is also another important factor in scaffold characterization, through which no significant differences in surface contact angles were observed among the various scaffold geometries. PLA, a material known for its hydrophilic properties, demonstrated the preservation of these characteristics after the 3D printing process. This was confirmed by measuring a static contact angle of 70.71 ± 2.52° across all scaffolds.

**FIGURE 2:**
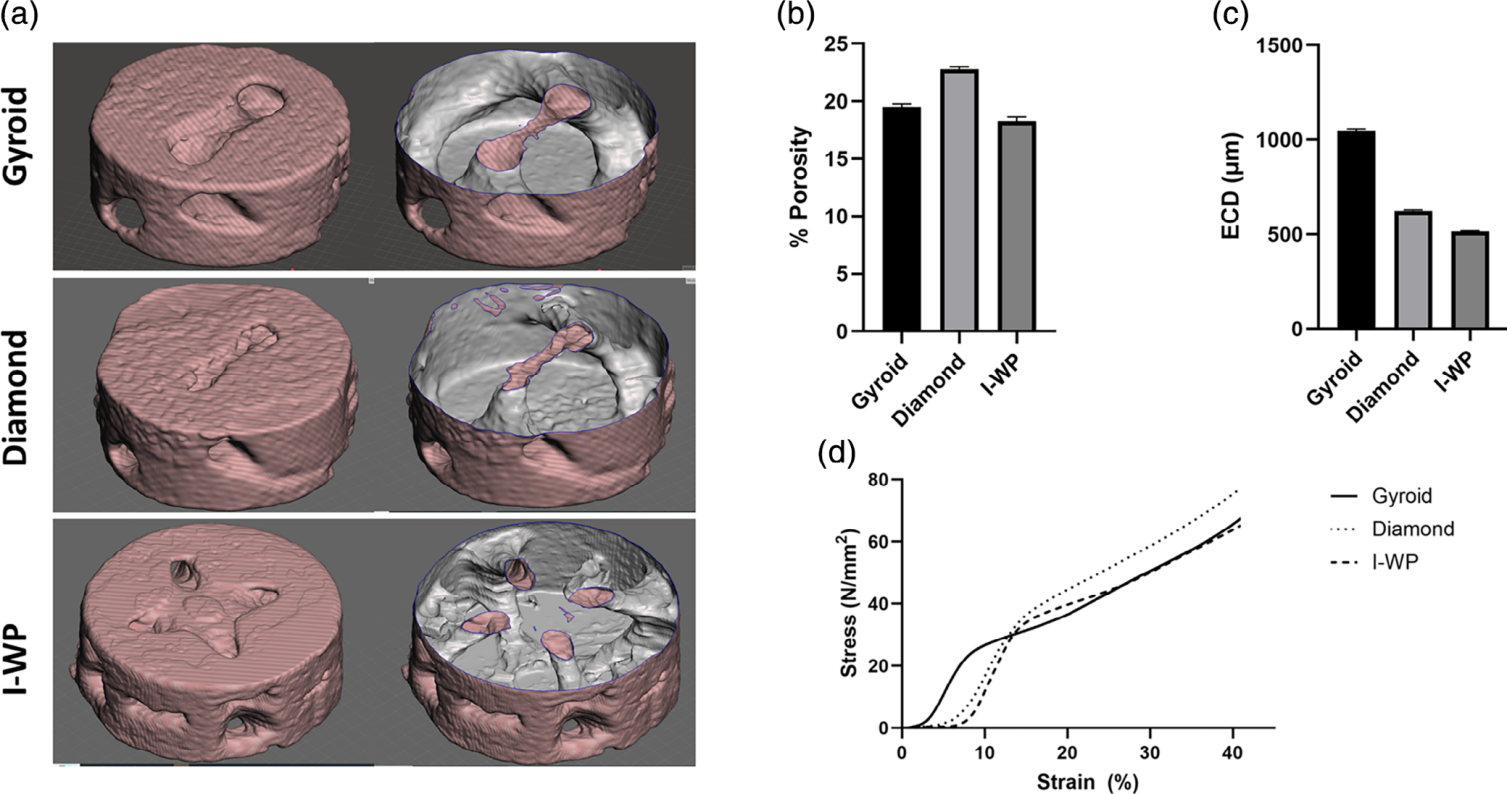
(a) Representative 3D reconstructions of Gyroid (top), Diamond (middle), and I‐WP (bottom) scaffolds from micro‐CT scans. The left column shows external morphology; the right column depicts internal porosity via transparent cut views. (b) Total porosity (%) based on binarized micro‐CT slices, with Diamond showing the highest values. (c) Equivalent Circular Diameter (ECD, μm) analysis indicating significantly larger pore size in Gyroid scaffolds. (d) Compressive stress–strain curves for each geometry showing typical nonlinear behavior. Diamond scaffolds exhibited the highest stiffness and peak stress; Gyroid showed the lowest. Values represent the mean trends from six specimens per group.

Micro‐CT imaging was first employed to visualize the internal porous architecture of the TPMS scaffolds, providing detailed cross‐sectional and cut‐view representations of their interconnected structures (Figure 2a). Quantitative microstructural analysis based on micro‐CT images revealed that the actual porosity values differed slightly from the designed porosity of 20%. Among the scaffold geometries, the diamond structure exhibited the slightly highest porosity, with a mean value of 22.76 ± 1.96%, followed by the gyroid (19.52 ± 2.24%) and IWP (18.27 ± 3.31%) designs (Figure 2b). These variations reflect minor deviations that commonly occur during the printing and post‐processing stages. In terms of pore size, the Equivalent Circular Diameter (ECD) analysis, also derived from micro‐CT image data, showed that the gyroid scaffold had the largest average pore diameter (1049.0 ± 104 μm), followed by the diamond (624.4 ± 56.61 μm) and IWP (519.9 ± 48.81 μm) scaffolds (Figure 2c). Unlike traditional porous structures defined by distinct voids, TPMS architectures are based on continuous surface curvature, and ECD serves as a practical 2D approximation of local pore openings within this complex geometry. These results demonstrate that scaffold geometry has a significant influence on both porosity and pore size, even when printed with identical nominal porosity settings.

The pressure stress–strain behavior of gyroid, diamond, and I‐WP lattice structures is shown in Figure 2d. All three structures displayed a typical nonlinear stress–strain response seen in cellular solids. This includes an initial linear elastic region, a plateau, and a densification phase. At 50% strain, the diamond structure had the highest compressive stress, averaging 98.39 N/mm^2^ with a standard deviation of 6.92. This result indicates its strong load‐bearing capacity. The gyroid structure followed with an average stress of 85.32 N/mm^2^ (SD: 6.68). The I‐WP structure recorded the lowest stress at 79.76 N/mm^2^ (SD: 1.56). The diamond and I‐WP lattices showed steeper initial slopes in their stress–strain curves. This suggests they have higher elastic moduli than the gyroid structure. So, the diamond and I‐WP configurations offer more stiffness during the initial elastic deformation phase. Overall, the diamond lattice excelled in stiffness and compressive stress at 50% strain, making it a strong candidate for load‐bearing biomedical applications.

### 4.2 Seeding capacity, cell attachment, and imaging

**FIGURE 3:**
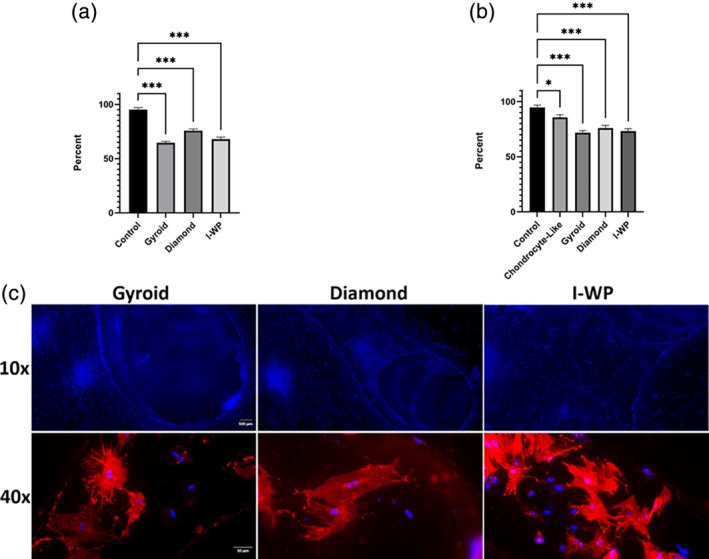
(a) Fluorescent images of Hoechst 33342 and Dil‐stained cells cultured on each scaffold type at 24 hours. The top row shows Hoechst‐stained stem nuclei at 10×, the second row shows merged images of Hoechst‐stained nuclei and Dil‐stained cell membrane on each geometry at 40×. (b) Assessment of initial cell attachment on each scaffold type at 24 h, expressed as the percentage of seeded cells; (c) Cell viability percentages after 21 days of chondrogenic differentiation on each scaffold type and 24‐well plate controls. Data are shown as arithmetic mean ± standard error (SEM). The asterisk (*) indicates statistical significance compared to the selected group (* = *p* < 0.05 ** = *p* < 0.01 *** = *p* < 0.001).

Cell seeding capacity was calculated as the absorbance percentage relative to 2 × 10^5^ cells seeded per 24‐well plate. Scaffold seeding capacity and adhesion rates were assessed after 24 hours using the CCK‐8 test. The cell attachment percentages of the adhered cells were determined to be: Gyroid 64.54 ± 1.43%, Diamond 75.75 ± 1.60%, and I‐WP 67.86 ± 1.95%. These findings indicate that the Gyroid and I‐WP geometries have a similar cell seeding capacities, whereas the Diamond geometry exhibits a better attachment rate (Figure 3a). Twenty‐four hours after the cell seeding, each scaffold was stained with Hoechst 34442 and Dil stains (Figure 3c). In the upper row, the largely homogeneous distribution of cell nuclei stained with Hoechst 33342 in blue on the inner and outer surfaces of the cell scaffold demonstrates the success of the dynamic seeding process. The uniformity and intensity of the staining indicate effective cell seeding and attachment, with slight variations potentially reflecting differences in scaffold geometry or surface imperfections. The bottom row showcases cellular interactions using the DiIC 18 (3) stain (red), which highlights cell membranes, overlaid with blue‐stained nuclei from Hoechst 33342. Cellular attachment observed across all geometries reveals a consistent cell attachment and spreading pattern on the scaffold surfaces. To examine how scaffold architecture influences cell proliferation during the differentiation period, we conducted a 21‐day viability assessment. The Control and Chondrocyte‐like cell groups were seeded in standard 24‐well plates as before and maintained viability levels of 94.68% ± 2.15 and 85.63% ± 2.26, respectively. In contrast, cells cultured on all scaffold geometries viability appears to be slightly lower. Among the TPMS‐based scaffolds, the Diamond geometry led with 75.92% ± 2.46. Following closely were the I‐WP at 73.27% ± 2.25 and the Gyroid at 71.80% ± 1.92 (Figure 3b). Although all types of scaffolds sustained viable cell populations throughout the 21‐day chondrogenic differentiation, their viability levels fell notably short of those seen in the 2D‐cultured Control and Chondrocyte‐like groups.

### 4.3 Examination of chondrogenic markers with RT‐PCR and Western blot

**FIGURE 4:**
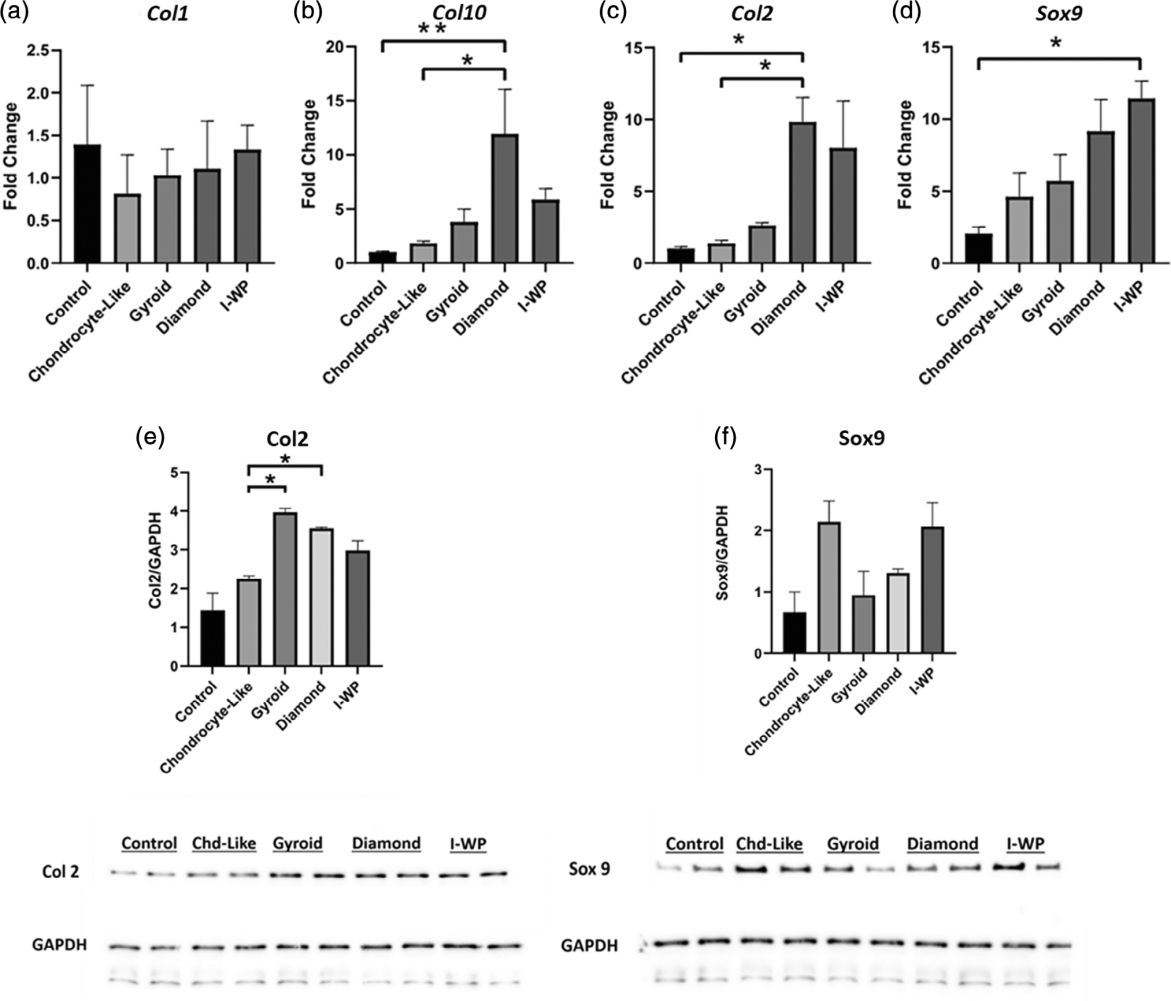
Relative gene expression levels of each scaffold geometry. (a) Collagen Type 1, (b) Collagen Type 10, (c) Collagen Type 2, (d) Sox 9. The results of triplicates are given as arithmetic mean ± standard error. The asterisk (*) indicates statistical significance compared to the selected group (*p* ≤ 0.05). Relative protein levels of each scaffold geometry. Collagen Type 2(Left), Sox 9 (right). The results of a duplicates are given as arithmetic mean ± standard error. The asterisk (*) indicates statistical significance compared to the selected group (p ≤ 0.05).

In all experiments, chondrogenically differentiated stem cells (chondrocyte‐like group) cultured on well plates served as a positive control to assess the impact of scaffold geometry on differentiation, with all data normalized to undifferentiated adipose stem cells (control group). *Col1* mRNA level showed a slight difference between the groups; this difference was not statistically significant, showing scaffold‐dependent variability ranging from a 0.81 to 1.33‐fold increase (Figure 4a). In contrast, mRNA levels of hypertrophic cartilage‐associated *Col10* gene varied between scaffolds of different geometries (Figure 4b). The chondrocyte‐like cell group showed a 1.83 ± 0.2‐fold change, the gyroid group 3.80 ± 1.19, the diamond group 11.95 ± 4.81, and the I‐WP group a 5.86 ± 1.01‐fold change (Figure 4b). mRNA levels of *Col2*, a critical marker of hyaline cartilage, were significantly increased in all scaffold geometries, with the highest increase (∼10‐fold) observed in the Diamond geometry; this was followed by I‐WP (∼8‐fold) and Gyroid (∼2.5‐fold) geometries, respectively (Figure 4c). In the Western blot analysis, protein levels of Col2 showed the following fold changes: the chondrocyte‐like cell group exhibited a 2.25 ± 0.07‐fold increase, the gyroid group 3.96 ± 0.10, the diamond group 3.55 ± 0.02, and the I‐WP group 2.97 ± 0.25 (Figure 4e).

*Sox9*, the chondrocyte‐like cell group displayed a 4.63 ± 1.66‐fold change, the gyroid group 5.70 ± 1.84, the diamond group 9.15 ± 2.20, and the I‐WP group an 11.43 ± 1.19‐fold change (Figure 4d). In the Western blot analysis for Sox9 protein levels across different TPMS scaffolds is presented in Figure 4f. Sox9 protein level, no significant differences were observed: the chondrocyte‐like cell group showed a 2.14 ± 0.33‐fold change, the gyroid group 0.94 ± 0.38, the diamond group 1.31 ± 0.06, and the I‐WP group a 2.06 ± 0.39‐fold change.

## 5 DISCUSSION

In tissue engineering practices, surface topography of the scaffolds is a crucial parameter to determine the cell adhesion, proliferation, and differentiation.^28^, ^29^ An examination of topographic features with electron microscopy and surface wettability of all scaffold geometries (Gyroid, Diamond and I‐WP) revealed similar physical environments at the microscale level (Figure 1). It was also observed that seeded cells successfully attached and showed a spreading pattern due to dynamic seeding on all scaffold surfaces (Figure 3c). Substrates with topographical features smaller than the size of a cell (<50 μm) have been shown to effectively enhance cell adhesion and spreading.^30^ Furthermore, as previously mentioned, cells can recognize surface topographies ranging from the nanometer scale to several hundred micrometers. They tend to exhibit preferences for disordered surfaces, which significantly influence their behavior compared to smooth, even surfaces.^31^ Like surface topography, hydrophilicity is another crucial factor that can significantly influence cellular characteristics, including proliferation, differentiation, and migration.^28^ Similar trends regarding the static contact angle of PLA have been observed before.^32^ Altogether, these results demonstrate that our 3D‐printed TPMS scaffolds provide a favorable biophysical environment at the cellular level, supporting early cell adhesion and spreading. Based on both literature findings and our initial observations, the surface microarchitecture and scaffold wettability appear to contribute to these cellular responses, reinforcing their suitability for tissue engineering applications.

In PLA scaffolds produced using the FDM technique, the dimensional gap between designed and actual porosity as well as pore size can be significant. This discrepancy often arises from the constraints of the extrusion process and printer resolution. Therefore, printed support structures frequently emerge thicker and with smaller pores than originally intended.^33^ For example, one of the studies aimed for a support width of 400 μm and pore sizes of 800 μm. However, the printed supports measured around 413 μm thick—3.2% thicker—while the pores shrank to approximately 739 μm, a reduction of 7.6%. This discrepancy led to a porosity value about 4–5% lower than the design.^33^ In a separate analysis by Buj‐Corral et al., the results indicated a porosity of 59.4% and an average pore size of 372 μm, contrasting with the designed targets of 53.7% porosity and 434 μm pore size.^34^ Here, the pore diameter exhibited a 14% decrease while porosity experienced a slight increase. These examples illustrate a crucial aspect of PLA scaffolds printed with FDM: deviations in pore size can typically vary between 5% and 15%, and changes in porosity may fluctuate by several percentage points.^33^, ^34^ In our case, porosities of the Gyroid, Diamond, and I‐WP geometries produced using the FDM method demonstrated acceptable dimensional 2–5%shrinkage in all samples, consistent with the tolerance ranges reported in the literature (Figure 2b). This supports both the validity and repeatability of the obtained data and demonstrates the reliability of the production method used.

TPMS structures have continuous, periodic surfaces that form interconnected porous networks. Unlike traditional foam or strut‐based scaffolds, they lack clear pore boundaries, requiring a different perspective in both structural analysis and biological interpretation. This makes traditional geometric measures, like minimum and maximum diameter, insufficient. To standardize, we used the Equivalent Circular Diameter (ECD) from 2D micro‐CT images. ECD helps simplify the complexity of TPMS. It offers a practical estimate of local pore openings in 2D projections. This aligns with established methods for irregular porous media.^35^, ^36^ For example, the higher ECD in the Gyroid structure means these pores have larger openings on average (Figure 2c). In contrast, the lower ECD in Diamond and I‐WP shows smaller and more tightly packed pores (Figure 2c). This difference matters biologically, the ability of cells to enter, attach, or work in these pores depends on the void ratio and the physical shape of the pores, including size, connectivity, and morphology.

It is well established that cell–cell contact plays a crucial role in promoting stem cell differentiation and survival, emphasizing the importance of maximizing cell attachment and the number of cells on our scaffolds.^37^ In chondrogenic differentiation protocols, full cell confluency is essential as it enhances cell–cell interactions, supports, and creates a cartilage‐like microenvironment with dense cell clusters.^38^ The cell‐bearing capacity of scaffold shapes can greatly affect chondrogenic differentiation success. In our study, Diamond geometry had the best initial cell attachment after 24 hours. Fluorescence microscopy images with DAPI dye, taken 24 hours after seeding, demonstrated uniform cell attachment across the scaffold surfaces in all cases (Figure 3c). However, differences among all shapes were not statistically significant (Figure 3a). Similarly, after 21 days of growth, only small differences were noted (Figure 3b). This is likely because the 3D porous structure of the scaffolds can either limit or boost cell growth compared to 2D surfaces. Research indicates that cell scaffolds with large pores (>1000 μm) lead to low cell adhesion initially. In contrast, medium‐sized pores (~500 μm) enhance cell attachment by increasing surface area and trapping cells found that 500 μm pores improved osteoblast colonization.^39^, ^40^ However, 1000 μm pores showed poor initial adhesion, even with vascularization.^40^ These results suggest that large pores in the gyroid may hinder early cell adhesion. Pores sized 700–1000 μm help with nutrient and oxygen diffusion and later vascularization. Barba et al. emphasized that pore sizes of 300–800 μm are crucial for bone growth and new blood vessel formation.^41^, ^42^ The gyroid's large porous structure supports these long‐term infiltration benefits. The initial cell attachment ratios show that Diamond (75%) is greater than I‐WP (68%), which is greater than Gyroid (64%). This highlights how microarchitecture affects cell retention. Although all scaffolds had the same porosity, the significantly larger ECD values of Gyroid (reflecting larger pore openings) were associated with a 24 h cell adhesion disadvantage (compared to 75.75% ± 1.60% of Diamond and 67.86% ± 1.95% of I‐WP), indicating a trade‐off between pore size and initial cell anchorage.

Twenty‐one‐day chondrocyte differentiation protocol was applied to adipose stem cells attached to Diamond, Gyroid, and I‐WP scaffolds, respectively, and its effect on the mRNA levels of key chondrocyte markers between groups was evaluated by RT‐PCR method.^27^ *Col1* is primarily a marker for fibrous connective tissue, bone, skin, and dentin, but is generally absent in hyaline cartilage. Overall, the fact that *Col1* mRNA levels, which are associated with fibrotic cartilage formation, did not show a statistically significant difference between the groups and remained relatively constant indicates that all scaffold geometries used effectively suppressed fibrotic tissue formation.^43^, ^44^ *Col10* is a well‐known marker of chondrogenic hypertrophy, playing a crucial role in cartilage‐to‐bone replacement during embryogenesis. While the Diamond group caused a significant increase in *Col10*, interestingly, the I‐WP and Gyroid groups did not show a similar increase. However, its expression is not desirable during chondrogenic differentiation, as it may indicate an unwanted shift toward hypertrophic changes.^45^, ^46^ These findings suggest that the Diamond geometry may induce undesired hypertrophic changes, whereas the I‐WP and Gyroid geometries may support a more stable chondrocyte phenotype compared to it.^44^, ^47^ In conclusion, all scaffold geometries exhibit higher *Col10* levels compared to stem cells and chondrogenic cells, which may be interpreted as an undesirable hypertrophic response present in all conditions.^47^ Among all scaffold geometries, Gyroid showed the lowest hypertrophic marker levels. One of the most important chondrogenic markers, *Col2* mRNA, also showed differences between the groups, with Diamond and I‐WP geometries resulting in the most pronounced fold changes. This suggests that Diamond and I‐WP geometries may provide the optimal microenvironment to promote enhanced *Col2* expression in mRNA levels. Although *Col2* mRNA levels showed a significant upregulation in these two scaffold geometries, protein levels do not collaborate with mRNA levels. Western blot analysis revealed that the Gyroid scaffold exhibited the highest Col2 protein levels, followed by the Diamond and I‐WP scaffolds (Figure 4c and Figure 4e). A similar discrepancy is also present in the gene expression and protein levels of Sox9, another critical marker of chondrocyte differentiation. When examined, an increase is observed in the Gyroid, Diamond, and I‐WP groups, respectively (Figure 4d). *Sox9* is an important regulator of *Col2* expression and maintains chondrogenic commitment of the differentiated cells. Coordination in the mRNA and protein levels of these important cartilage markers is crucial to produce cartilage‐specific extracellular matrix and stable cartilage tissue.^48^, ^49^ A significant increase in mRNA levels was observed in the Diamond and I‐WP groups, respectively (Figure 4d). Among these, the I‐WP group showed the most pronounced and statistically significant rise in *Sox9* mRNA expression, despite all TPMS geometries leading to a noticeable upregulation. However, the Sox9 protein level, although there was no statistically significant change between the groups, a decrease of nearly half was detected, especially in the Gyroid and Diamond groups (Figure 4).

According to our results, Diamond and I‐WP geometries exhibit consistently high *Col2* and *Sox9* gene expression levels while also maintaining elevated *Col10* mRNA levels, which may indicate hypertrophic involvement. The variation in the hypertrophic marker (*Col10*) among the scaffolds suggests potential differences in their ability to promote endochondral bone formation. Meanwhile, while Gyroid geometry shows a lower mRNA level among the groups, elevated Col2 protein levels and suppressed Sox9 could be interpreted as a more stable chondrogenic differentiation environment among the applied scaffold geometries. Although significant differences in mRNA and protein levels of Col2 and Sox9 are observed between the groups, as previously stated, mRNA expression does not always directly translate to protein production. Therefore, we consider Western blot results to be more reliable for interpreting the effects of different geometries on chondrogenesis in vitro.^50^ It has been suggested that the TPMS scaffold geometries used in our study can be designed to mimic the mechanical properties of jawbone, cortical bone, and cancellous bone, and can be supported with modifications to facilitate cell survival and attachment.^51^ Interestingly, in vitro studies on TPMS scaffolds with similar geometries have reported no significant geometry‐dependent differences in the gene expression levels associated with osteogenic differentiation in bone marrow stem cells between Gyroid and Diamond structures. However, in vivo studies on TPMS scaffolds with Gyroid and Diamond microstructural structures have demonstrated enhanced bone tissue regeneration when in direct contact with bone.^52^ In another similar in vitro study, it was observed that Diamond, Gyroid, and I‐WP geometries could increase Runx2 and OPN gene expression levels, which are markers of osteogenic differentiation, by more than 2‐fold, and in vivo applications, again, Gyroid and Diamond geometries made a significant contribution to tissue formation.^53^ These differences depend on geometry and relate to local pore size and curvature, which affect cellular environments. Larger pore openings, like those in Gyroid scaffolds, help nutrients and oxygen diffuse better. They also assist in waste removal, supporting long‐term chondrogenic activity.^54^, ^55^ However, these larger spaces may provide fewer anchorage points for cells during early adhesion. This could lower cytoskeletal tension and impact early differentiation signals.^56^ On the other hand, the smaller, tighter pores in Diamond and I‐WP scaffolds can create localized hypoxic areas and encourage greater cell condensation. This is known to boost Sox9 expression and early chondrogenic commitment.^57^, ^58^ The tight pore geometry can also improve cell‐ECM contact and enhance focal adhesion signaling. This may explain the stronger initial mRNA upregulation seen in those groups.

## 6 CONCLUSION

Through the analysis of cartilage markers (Col2, Sox9) and hypertrophic markers (*Col10*, *Col1*), our research concludes by demonstrating the important influence of scaffold shape on chondrogenesis. Significant differences were seen in the mRNA and protein expression profiles, even though all three TPMS geometries—Diamond, Gyroid, and I‐WP—supported efficient cell attachment and early chondrogenic development. The Gyroid scaffold showed better Col2 protein production and a more stable chondrogenic phenotype with lower hypertrophic signaling, as evidenced by decreased Col10 expression, whereas the Diamond and I‐WP scaffolds promoted high levels of chondrocyte‐specific mRNA markers (*Col2* and *Sox9*). These discrepancies imply that protein production and stability may be modulated by scaffold‐specific effects and post‐transcriptional regulatory mechanisms.

In conclusion, Gyroid scaffolds appear optimal for long‐term cartilage stability due to their ability to promote chondrogenic markers while minimizing hypertrophy. I‐WP scaffolds also show promise, whereas Diamond scaffolds may require modifications to reduce hypertrophic risks. Future research should investigate mechanical stimulation or biochemical enhancements to optimize these scaffold designs for cartilage regeneration. While Gyroid scaffolds may facilitate cell infiltration and nutrient diffusion, Diamond and I‐WP architectures, with their smaller and more confined pore morphologies, appear to support more robust expression of hyaline cartilage markers, potentially due to enhanced cell condensation and mechanical microenvironments favoring chondrogenic signaling.

## AUTHOR CONTRIBUTIONS

Mahmut Alp Kılıç and Mustafa Akyürek: Cell culture, in vitro assays, writing. Alp Karakoç and Roozbeh Abidnejad: Scaffold design, manufacturing, imaging, writing.

## FUNDING INFORMATION

This study was supported by the Scientific Research Projects Unit of Canakkale Onsekiz Mart Universitesi with the project number “TSA‐2021‐3790”.

## CONFLICT OF INTEREST STATEMENT

The authors declare that there is no conflict of interest regarding the publication of this paper. Funding for this research was provided by Research Projects Unit of Canakkale Onsekiz Mart Universitesi, which had no role in the study design, data collection and analysis, decision to publish, or preparation of the manuscript.

## Supporting information


**Figure S1.** Schematic representation of the scaffold manufacturing process, illustrating the step‐by‐step procedure from initial material preparation to the final scaffold structure. b. Image of the manufactured scaffold, showcasing the intricate design and structural integrity.
**Figure S2.** Dynamic seeding method utilized as shown in figure. Sterilized, fibronectin‐coated scaffolds were placed in glass vials. Cells were then seeded directly onto the scaffolds, and the vials were left undisturbed at room temperature for 10 minutes to allow for initial cell attachment before being transferred to an incubator.
**Figure S3.** Alcian blue staining of a 21‐day chondrocyte differentiation protocol in plates. Undifferentiated cells showed faint blue staining (left), while differentiated cells exhibited strong staining areas, indicating successful chondrocyte‐like differentiation.
**Figure S4.** Relative gene expression levels of control and chondrocyte differentiated cells. The results of triplicates are given as arithmetic mean ± standard error. The asterisk (*) indicates statistical significance compared to the selected group (*p* ≤ 0.05).

## Data Availability

The data that support the findings of this study are available from the corresponding author upon reasonable request.
