# Supplementary material for: Evaluation of triply periodic minimal surface geometries in 3D‐printed PLA scaffolds for chondrogenic differentiation
Source: Biotechnol Prog. 2025 Aug 11;41(6):e70062. doi: 10.1002/btpr.70062 (PMC12696442; doi:10.1002/btpr.70062)
Supplement: Supplementary file 1 — Figure S1. Schematic representation of the scaffold manufacturing process, illustrating the step‐by‐step procedure from initial material preparation to the final scaffold structure. b. Image of the manufactured scaffold, showcasing the intricate design and structural integrity. Figure S2. Dynamic seeding method utilized as shown in figure. Sterilized, fibronectin‐coated scaffolds were placed in glass vials. Cells were then seeded directly onto the scaffolds, and the vials were left undisturbed at room temperature for 10 minutes to allow for initial cell attachment before being transferred to an incubator. Figure S3. Alcian blue staining of a 21‐day chondrocyte differentiation protocol in plates. Undifferentiated cells showed faint blue staining (left), while differentiated cells exhibited strong staining areas, indicating successful chondrocyte‐like differentiation. Figure S4. Relative gene expression levels of control and chondrocyte differentiated cells. The results of triplicates are given as arithmetic mean ± standard error. The asterisk (*) indicates statistical significance compared to the selected group (p ≤ 0.05). [file BTPR-41-e70062-s001.pdf]

Supplementary Material Description

Four figures that highlight significant facets of the experimental investigation are included in the supplemental material. The cell seeding technique is covered in Supplement Fig. 1(a) Schematic representation of the scaffold manufacturing process, illustrating each step from initial material preparation to the final 3D-printed structure. The workflow includes material formulation, CAD modeling of TPMS geometries, slicing, FDM-based printing, and post-processing steps to obtain structurally stable and sterile scaffolds. (b) A representative photograph of the fabricated PLA scaffold highlights the structural fidelity and geometric complexity of the printed lattice, confirming the successful translation from digital design to physical object. Supplement Fig. 2 uses Alcian blue staining to indicate that cells were successfully chondrogenic differentiation using a 21-day regimen. Differentiated cells exhibit strong staining, indicating the growth of chondrocyte-like cells, while undifferentiated cells show faint staining. Supplement Fig. 3, which shows relative gene expression levels obtained using the PCR technique, provides additional evidence for chondrogenic differentiation. The results show statistically significant differences from the control group, indicated by the arithmetic mean ± standard error from triplicates. Supplement Fig. 4 shows two Western blot analyses. The first one (a) is for Sox9 expression and the second one (b) is for Col 2 expression. Both analyses are conducted on different scaffold types: Control, Chd-Like, Gyroid, Diamond, and I-WP. GAPDH is used as a loading control in both experiments to ensure that the amount of protein loaded in each lane is comparable.

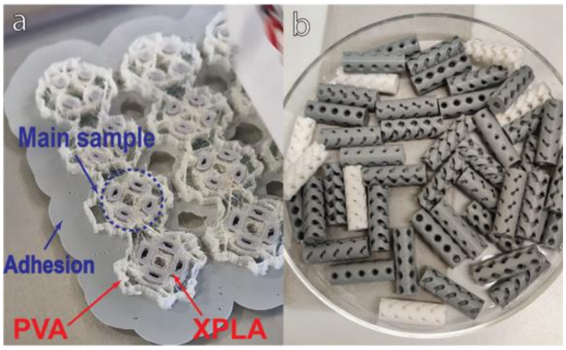

Supplementary Figure 1. Schematic representation of the scaffold manufacturing process, illustrating the step-by-step procedure from initial material preparation to the final scaffold structure. b. Image of the manufactured scaffold, showcasing the intricate design and structural integrity.

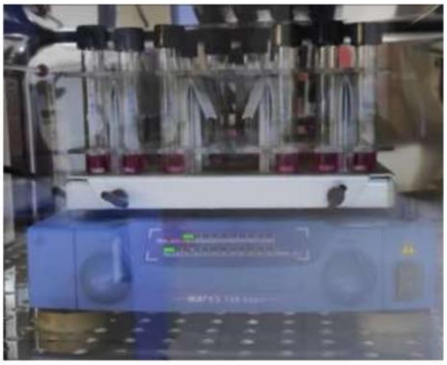

Supplementary Figure 2. Dynamic seeding method utilized as shown in figure. Sterilized, fibronectin-coated scaffolds were placed in glass vials. Cells were then seeded directly onto the scaffolds, and the vials were left undisturbed at room temperature for 10 minutes to allow for initial cell attachment before being transferred to an incubator.

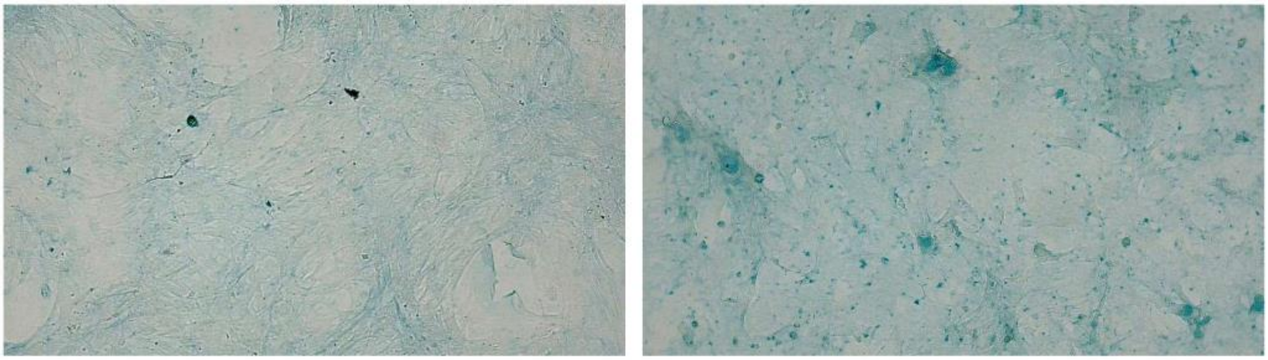

Supplementary Figure 3. Alcian blue staining of a 21-day chondrocyte differentiation protocol in plates. Undifferentiated cells showed faint blue staining (left), while differentiated cells exhibited strong staining areas, indicating successful chondrocyte-like differentiation

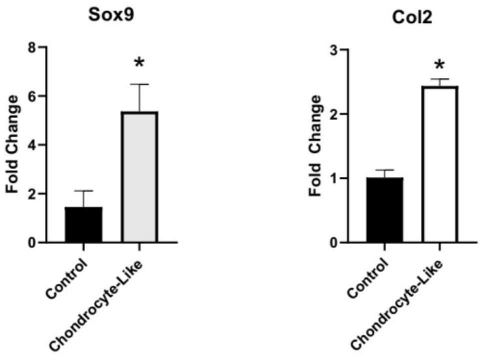

Supplementary Figure 4. Relative gene expression levels of control and chondrocyte differentiated cells. The results of triplicates are given as arithmetic mean ± standard error. The asterisk (*) indicates statistical significance compared to the selected group ($p \leq 0.05$).
